# Pharmacokinetic Profile of Brepocitinib with Topical Administration in Atopic Dermatitis and Psoriasis Populations: Strategy to Inform Clinical Trial Design in Adult and Pediatric Populations

**DOI:** 10.1007/s11095-024-03654-w

**Published:** 2024-03-22

**Authors:** Farzaneh Maleki, Cheng Chang, Vivek S. Purohit, Timothy Nicholas

**Affiliations:** Clinical Pharmacology & Pharmacometrics, Global Product Development, Pfizer, Cambridge, MA 02139 USA

**Keywords:** atopic dermatitis, brepocitinib, population pharmacokinetics, psoriasis, topical drug

## Abstract

**Introduction:**

Topical brepocitinib, a tyrosine kinase (TYK)2/Janus kinase (JAK)1 inhibitor, is in development for psoriasis (PsO) and atopic dermatitis (AD). Quantitative analyses of prior clinical trial data were used to inform future clinical trial designs.

**Methods:**

Two phase 2b studies in patients with AD and PsO were used to characterize the amount of topical brepocitinib and the resultant systemic trough concentration (*C*_Trough_) using a linear mixed-effects regression (LMER). This model was used to predict brepocitinib systemic *C*_Trough_ for higher treated body surface areas (BSAs) in adults and children. Information from non-clinical and clinical trials with oral brepocitinib was leveraged to set safety thresholds. This combined approach was used to inform future dose-strength selection and treated BSA limits.

**Results:**

Data from 256 patients were analyzed. Patient type, dose strength, and frequency had significant impacts on the dose–exposure relationship. Systemic concentration in patients with PsO was predicted to be 45% lower than in patients with AD from the same dose. When topically applied to the same percentage BSA, brepocitinib systemic exposures are expected to be comparable between adults and children. The systemic steady-state exposure after 3% once daily and twice daily (2 mg/cm^2^) cream applied to less than 50% BSA in patients with AD and PsO, respectively, maintains at least a threefold margin to non-clinical safety findings and clinical hematologic markers.

**Conclusion:**

The relationship between the amount of active drug applied and brepocitinib systemic *C*_Trough_, described by LMER, may inform the development strategy for dose optimization in the brepocitinib topical program.

**Supplementary Information:**

The online version contains supplementary material available at 10.1007/s11095-024-03654-w.

## Introduction

Atopic dermatitis (AD), also known as atopic eczema, is a common, chronic, inflammatory skin disorder affecting up to 2.4% of the population worldwide and is characterized by dry, red skin lesions and intense pruritus [1, 2]. Prevalence estimates suggest that approximately 15–20% of children [3] and 5–10% of adults worldwide suffer from AD [4]. The onset of AD generally occurs in early childhood, which results in a reduced quality of life [1]. Multiple cytokines are implicated in AD, including tyrosine kinase (TYK)2-dependent interleukin (IL)-12, IL-23, IL-4, IL-13, IL-22, IL-31, and thymic stromal lymphopoietin (TSLP), which all signal via Janus kinase (JAK1) [5–7]. Inhibition of the JAK family has been shown to improve AD symptoms [4, 8, 9].

Psoriasis (PsO) is an inflammatory skin disease characterized by red patches covered with white scales [10]. Chronic plaque PsO is the most common chronic dermatitis, with a reported prevalence ranging between 0.09% and 11.43% [11], and is estimated to afflict more than 7.5 million people in the US and roughly 125 million people worldwide [12]. Although PsO primarily affects the skin and is not a life-threatening disease, it can profoundly impact a patient’s health-related quality of life and could lead to a greater risk of developing conditions such as type 2 diabetes, myocardial infarction, and psoriatic arthritis (PsA) [13–15]. IL-17A and IL-23 play an important role in the pathology of PsO [16, 17]. Biologics modulating IL-23 and IL-17 have shown efficacy and are approved for use in PsO [16, 18]. Inhibition of TYK2 and JAK1 inhibits the IL-23/IL-17 axis, which could result in improved PsO symptoms [19].

Brepocitinib (PF-06700841) is a potent dual TYK2/ JAK1 inhibitor that has shown promising results in improving signs and symptoms of dermatological diseases, such as AD and PsO [20, 21]. Brepocitinib, as an orally bioavailable small molecule, has also been investigated for the treatment of systemic lupus erythematosus, PsO, Crohn’s disease, ulcerative colitis, PsA, alopecia areata, vitiligo, hidradenitis suppurativa, non-infectious uveitis, and dermatomyositis [21–23]. A topical formulation of brepocitinib has been developed for the treatment of PsO and AD.

An understanding of the systemic concentration is critical in the development of topical drugs for dose-strength selection, since high levels of systemic exposure can cause safety concerns, especially in the pediatric population. It is expected that with topical treatment, the systemic exposure level is generally lower than with oral treatment. Administered topical dose levels can vary by treated body surface area (BSA) and drug application rate. Topical drug formulations differ in physicochemical properties, which can impact the pharmacokinetics (PK) of the drug (absorption, distribution, metabolism, and elimination) [24]. Topical formulations may impact the skin barrier differently in different diseases, disease severities, and age groups [24, 25]. Pediatric systemic concentrations could differ from those of adults due to higher surface area to volume ratio, resulting in increased absorption and differences in clearance [26].

The goal of this study was to determine optimal brepocitinib dosing for future clinical trials. In this paper, we describe quantitative approaches used to characterize the relationship between the amount of brepocitinib administered topically and the resultant systemic trough concentrations (*C*_Trough_) in patients with PsO and AD. We also demonstrate how to leverage data from non-clinical and clinical studies to inform future clinical trial design in adults, as well as in the pediatric population.

## Materials and Methods

### Modeling Framework

**Fig. 1:**
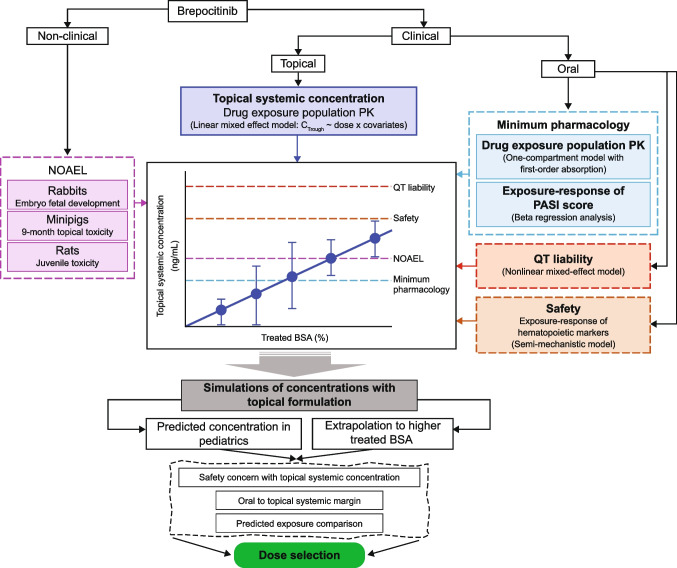
Modeling framework. *BSA*, body surface area; *C*_Trough_, trough concentration; *NOAEL*, no observed adverse effect level;* PASI*, Psoriasis Area and Severity Index; *PK*, pharmacokinetics.

Information from multiple modeling and simulation components was leveraged to conduct this analysis (see Fig. 1). Using clinical topical data, a relationship between the amount of brepocitinib and the systemic *C*_Trough_ was established. Non-clinical and clinical oral studies (previous modeling and simulation work) were then used to set safety thresholds for systemic concentrations. The topical model was used for simulation of concentration in the pediatric population and extrapolation to higher treated BSA in adults, which, along with the safety threshold, enabled us to select a dose strength and the maximum safely percentage treated BSA, which has the best balance between safety and efficacy.

### Brepocitinib Clinical Studies with Topical Administration

#### Pharmacokinetic Assessments

PK assessments from two Phase 2b, randomized, double-blind, vehicle-controlled, parallel-group, dose-ranging studies were used to conduct this analysis. Brepocitinib was administered topically once daily (QD) or twice daily (BID) at multiple dose strengths in patients with mild or moderate AD (NCT03903822), or with mild to moderate chronic PsO (NCT02969018). Patients were randomized into vehicle and active treatment groups with dose strengths of 0.1%, 0.3%, 1%, or 3%. The study durations were 6 and 12 weeks for AD and PsO patients, respectively. The target dose application rate (APR) was predefined as 2 mg formulation per cm^2^ of treated BSA. The maximum tested BSA was 22% in patients with AD, which was associated with a predicted systemic margin of ~ 1.9-fold following topical application of the highest dose strength (3%; the predicted margins relative to the exposure achieved following oral administration of 30 mg QD).

Trough plasma samples were collected pre-dose on Days 1, 8, 15, 22, 29, and 43 for the AD population and pre-dose on Days 1, 8, 15, 29, 43, 57, 71, and 85 for the PsO population. Screening observations and missed doses were excluded from the analysis. Data records with missing *C*_Trough_ or zero percentage treated BSA were excluded from the analysis. Observed *C*_Trough_ below limit of quantification (BLQ) were also excluded from the analyses. A summary of the clinical studies included in this analysis is presented in Table S1 of the Online Resource.

#### LMER Model Development

A linear mixed-effect regression (LMER) analysis was conducted to investigate the relationship between the amount of active drug administered topically and systemic *C*_Trough_:

1 $${C}_{{\text{Trough}}}={\text{Int}}+{\text{Slope}} \cdot {\left(\frac{{{\text{BWT}}}_{i}}{70}\right)}^{-0.75}\cdot {{\text{AMT}}}_{{\text{Drug}}}$$

where *C*_Trough_ is the systemic trough concentration in ng/mL, and Int is the intercept, which was fixed at 0 based on the expectation of no systemic exposure without treatment. Slope, which corresponds to the reciprocal of Clearance (Slope = bioavailability/clearance*dosing frequency interval), is the average slope for patients, and AMT_Drug_ is the amount of active drug in mg [27]. The effect of a baseline body weight (BWT) was included as the power function with a fixed exponent of –0.75 and referenced to 70 kg (healthy white male weight). The amount of active drug is derived by multiplying the amount of cream being applied by dose strength. For example, for a 2% strength cream (20 mg/g):

2 $${{\text{AMT}}}_{{\text{Drug}}}= 0.02\cdot {{\text{AMT}}}_{{\text{Cream}}}$$

where the amount of cream (mg) applied per dose is calculated as:

3 $${\text{AMT}}_\text{Cream}=\frac{\mathrm{Amount}\;\mathrm{of}\;\mathrm{cream}\;\mathrm{disposed}-\mathrm{Amount}\;\mathrm{of}\;\mathrm{cream}\;\mathrm{returned}}{\mathrm{Number}\;\mathrm{of}\;\mathrm{actual}\;\mathrm{doses}}$$

It should be noted that the amount of cream is the average amount of cream applied per dose over the dispensing interval (from the start to the end of treatment). The amount of brepocitinib applied for topical application depends on the BSA affected by the disease (i.e., AD or PsO). The APR per dose is related to both the amount of cream applied per dose and the treated BSA:

4 $${{\text{APR}}}_{{\text{Cream}}}= \frac{{{\text{AMT}}}_{{\text{Cream}}} }{{{\text{BSA}}}_{{\text{Treated}}}}$$

Patients were instructed on how much cream to apply to achieve the target APR of 2 mg/cm^2^.

This approach has also been used in topical crisaborole and tofacitinib programs [27, 28]. The inter-individual variability (IIV) on the parameter Slope was added using a log-normal distribution:

5 $${{\text{Slope}}}_{i}={\theta }_{{\text{Slope}}}\cdot {e}^{{\eta }_{i}}$$

where ${\eta }_{i}$ is a normal distribution with a mean of 0 and a variance of ${\omega }^{2}$, ${{\text{Slope}}}_{i}$ is the individual value for the *i*th patient, and ${\theta }_{{\text{Slope}}}$ is the population typical value.

The residual variability was described using the log-transform combined error model:

6 $${{\text{DV}}}_{\mathit{ij}}={{\text{IPRED}}}_{\mathit{ij}}+\left(\sqrt{{\sigma }_{{\text{add}}}^{2}+{\left({\sigma }_{{\text{pro}}}\cdot {{\text{IPRED}}}_{ij}\right)}^{2}}\right)\cdot {\varepsilon }_{ij}$$

where ${{\text{DV}}}_{ij}$ and ${{\text{IPRED}}}_{ij}$ are the observed and predicted brepocitinib concentration for the *i*th individual at *j*th time, respectively. ${\varepsilon }_{ij}$ is a normally distributed error term with a fixed mean of 0 and a variance of 1. ${\sigma }_{{\text{add}}}$ and ${\sigma }_{{\text{pro}}}$ are the additive and proportional components of the residual error, respectively.

Once the structural and error models were established, categorical covariates were included using the stepwise covariate modeling approach (forward selection followed by backward elimination). The effect of a categorical covariate on the parameter Slope was represented as follows:

7 $${\mathit{Slope}}_i=\theta_{\mathit{Slope}}\cdot P_{COV}\;\;where\;\;P_{COV}=\left\{\begin{array}{c}1\;if\;COV=0\\1+\theta_{Cov}\;if\;COV=1\end{array}\right.$$

where ${\theta }_{{\text{Slope}}}$ is the typical value of the parameter Slope, COV is a categorical covariate, and ${\theta }_{{\text{Cov}}}$ is the parameter for the effect of the covariate on ${\theta }_{{\text{Slope}}}$. A covariate remained in the model if its addition resulted in a significant decrease in objective function value (OFV) as assessed by the likelihood ratio test (*p* < 0.01) compared with the nested model. In addition, the 90% confidence interval (CI) of the parameter estimate should not include zero, and the covariate results in a reduction in IIV on the target population parameter.

#### Model Evaluation

The models were evaluated based on changes in the minimum OFV and diagnostic plots of observed concentrations *vs*. population predictions (PRED) or individual predictions (IPRED), and normalized prediction distribution errors (NPDE) *vs*. time after first dose (TAFD). Normality of conditional weighted residuals distributions was tested by evaluating distribution density and quantile–quantile plots. Normality of η distributions (as described by the empirical Bayes estimate [EBE] for random-effect parameters) was tested by evaluating distribution density plots. A model’s condition number (the square root of the ratio of the highest and lowest eigenvalues) was used to evaluate candidate models for overparameterization, where models with condition numbers less than 100 were considered for further development. The precision of the parameter estimates was determined by calculating the relative standard error. The fixed- and random-effect estimates with relative precisions below 20% were considered as precisely estimated. η-shrinkage was evaluated for the validity of using EBE for model diagnosis. If shrinkage exceeded 20%, caution was exercised in the interpretation of individual parameters. The predictive performance of the final model was evaluated by a visual predictive check (VPC) and prediction-corrected visual predictive check (pcVPC) based on 1,000 simulations of the index dataset [29]. Subpopulations (by study, covariates, dosing regimen, etc.) were used to stratify a summary of the predictive performance.

#### Strategy and Software

The LMER model was developed in MonolixSuite version 2020R1 [30]. The stochastic approximation expectation-maximization algorithm (SAEM) was used for model optimization [31]. Log likelihood (logLik) was obtained using the linearization method. Post-processing of the Monolix output and diagnostic plots was carried out using R statistical and programming language [32]. The Monolix Model files are included in the Online Resource.

### Brepocitinib Clinical Studies with Oral Administration

**Fig. 2:**
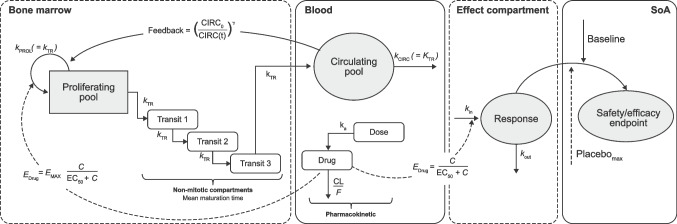
Schematic illustration of pharmacokinetic/pharmacodynamic models associated with oral brepocitinib treatment consisting of one compartment PopPK model with linear elimination and first-order absorption to describe the brepocitinib longitudinal systemic exposure [30]; semi-mechanistic Friberg models [35] to describe time profiles of reticulocyte and neutrophil counts after oral administration of brepocitinib; indirect response model with hyperbolic drug effects used for exposure–response analysis of PASI score in PsO patients; and indirect response model with hyperbolic drug effects to describe the relationship between brepocitinib systemic exposure and QTc prolongation after oral administration. *C,* concentration; *CIRC*, circulating drug; *CL*, clearance; *E*, effect; *EC*_*50*_, half maximal effective concentration; *F*, bioavailability; *PASI*, Psoriasis Area and Severity Index; *Placebo*_*max*_, maximum placebo response; *PsO*, psoriasis; *SoA*, site of action.

Multiple modeling and simulation works have been conducted to inform the decision-making process in clinical trials with oral brepocitinib treatment. Models are objects that evolve through the inclusion of greater information as part of their model development life cycle. As these models characterize exposure, safety, and efficacy relationships, they are typically utilized, re-utilized, and linked to gain a better quantitative understanding of the drug-development program. The models utilized for this assessment are listed below (see Fig. 2):

- Population PK model describing the brepocitinib exposure in different patient populations [33]
- Population exposure–response analysis characterizing the relationship between exposure and Psoriasis Area and Severity Index (PASI) scores in patients with PsO
- Population exposure–response models exploring the effect of oral brepocitinib administration on reticulocyte and neutrophil time profiles in different patient populations [34]
- Concentration-Fridericia-corrected QT (QTcF) model to characterize the relationship between brepocitinib systemic exposure and QTc prolongation after oral administration in healthy volunteers.

The above-mentioned models were used to provide a safety context for the topical program.

### Brepocitinib Non-Clinical (Toxicology) Studies

Non-clinical toxicology studies were conducted to support the clinical use of brepocitinib by the oral route of administration, including single- and repeat-dose oral toxicity studies in rats and monkeys. Pivotal embryo–fetal development studies (EFD) were also conducted in rats and rabbits. Topical administration of brepocitinib was tested in minipigs. The 0.1% dose strength was the lowest strength tested in *in vitro* systems and showed pharmacological activity in human skin that suggested it was the concentration at which we begin to observe loss of activity.

The PK of brepocitinib in *in vitro* studies was used to calculate the no observed adverse effect levels (NOAELs), which are associated with the maximum dose level at which no significant increase in adverse effects is observed compared with the vehicle group.

### Selection of Optimal Treatment Dose and Regimen

The final LMER model was used to support the Maximal Usage Trial (MUsT) strategy and phase 3 study design. The following criteria were considered for optimal dose and maximum treated BSA selection:

- Acceptable safety margins against non-clinical and clinical safety thresholds must be captured (threefold). Comparable systemic exposure to minimal systemic pharmacological activity level must also be observed
  - Extrapolation to higher treated BSA in adults: Clinical trial simulations were performed to project the systemic *C*_Trough_ at a given percentage treated BSA higher than the upper limit tested in phase 2b studies (22% and 15% in AD and PsO populations, respectively)
  - Calculation of oral to topical systemic margins: The projected systemic *C*_Trough_ was used to calculate safety margins against non-clinical NOAELs (*C*_ave_), clinical hematopoietic thresholds (IC_10_), QT liability (*C*_max_), and minimal systemic pharmacological activity (*C*_ave_) associated with the PsO population with oral brepocitinib treatment.
- The limitation of BSA in the younger age range must maintain safety margins (five-fold).
  - Extrapolation of concentrations to the pediatric population: The final model was also used to project the systemic exposure in pediatric patients. The amount of active drug being applied in a pediatric population was allometrically scaled with BWT with a fixed exponent of –0.75. The systemic exposure in pediatric patients down to 2 years of age is expected to be comparable with that in adult patients based on the first principles of clinical pharmacology, where, in pediatric patients, the reduction in drug clearance is canceled out by the reduction in BSA-based dose, resulting in comparable systemic exposure between pediatric and adult patients with the same percentage treated BSA [27, 28].
    8 $${\mathit{CL}}_{\mathit{Pediatrics}}= {CL}_{Adults}\cdot {\left(\frac{BW{T}_{pediatric}}{70}\right)}^{-0.75}$$

## Results

### Patient Characteristics in the Topical Clinical Studies

In total, there were 256 patients (118 patients with AD and 138 patients with PsO) with measured steady-state systemic *C*_Trough_ for brepocitinib. From a total of 2,997 available records, only 1,143 were used in the analysis. A total of 56% of records were BLQ and 5% had missing BSA data. The distribution of excluded observations was similar among treatment groups (dose-strength and frequency). However, more data were excluded from the PsO population (62%) than from the AD population (38%). Patient (median [range]) characteristics are detailed in Table 1. The population was predominantly white male with a median age of 45 years and BWT of 79 kg. The median (range) of administered active drug (drug amount normalized by dose strength) was 23.8 mg (0.1–501 mg).

**Table 1:** Summary of Demographics by Patient Type

	AD	PsO	Total
	Median [range]		
Age (years)	36.5 [17.0–74.0]	51.0 [18.0–75.0]	45.0 [17.0–75.0]
Baseline body weight (kg)	72.2 [47.5–128.0]	86.0 [47.6–143.0]	79.0 [47.5–143.0]
Treated BSA (%)	7.00 [1.00–22.0]	6.0 [2.0–15.0]	6.0 [1.0–22.0]
Application rate (mg/cm^2^)	1.60 [0.0475–25.4]	1.9 [0.3–7.91]	1.83 [0.0475–25.4]
Treatment group, *n* (%)			
0.1% QD	3 (2.2)	11 (9.3)	14 (5.5)
0.3% QD	11 (8.0)	14 (11.9)	25 (9.8)
1.0% QD	21 (15.2)	23 (19.5)	44 (17.2)
3.0% QD	30 (21.7)	26 (22.0)	56 (21.9)
0.3% BID	19 (13.8)	20 (16.9)	39 (15.2)
1.0% BID	26 (18.8)	24 (20.3)	50 (19.5)
3.0% BID	28 (20.3)		28 (10.9)
Sex, *n* (%)			
Female	41 (29.7)	59 (50.0)	100 (39.1)
Male	97 (70.3)	59 (50.0)	156 (60.9)
Race, *n* (%)			
White	114 (82.6)	68 (57.6)	182 (71.1)
Asian	19 (13.8)	32 (27.1)	51 (19.9)
African American	3 (2.2)	15 (12.7)	18 (7.0)
Other races	2 (1.4)	3 (2.5)	5 (2.0)

*AD*, atopic dermatitis; *BID*, twice daily; *BSA*, body surface area; *PsO*, psoriasis; *QD*, once daily

### Topical Dose–Systemic Exposure Relationship

The base LMER model with systemic *C*_Trough_ as the dependent variable and amount of active drug as the independent variable was defined with an estimated slope and intercept fixed to 0 (see Eq. 1). Covariates such as patient type and dose strength/regimen were tested on the parameter Slope. Parameter estimates and bootstrap results for the final model are shown in Table 2. The goodness-of-fit plots indicated an overall reasonable performance of the model (Fig. S1–S3).

**Table 2:** Parameter Estimates for the Final Model

Parameter	Value	Bootstrap median	Bootstrap CI (90%)	SHK (%)
–2 logLik	2,642			
Condition number	3.4			
Population parameter				
Slope^a^ (1/L)	0.0232	0.0238	(0.0177, 0.0322)	
Effect of moderate patients with PsO on slope	–0.449	–0.457	(–0.625, –0.200)	
Effect of twice daily dosing regimen on slope	0.564	0.614	(0.225, 1.20)	
Effect of 0.1% dose strength on slope	6.38	5.31	(2.99, 8.80)	
Effect of 1.3% dose strength on slope	2.91	2.79	(1.73, 4.42)	
Effect of 1.0% dose strength on slope	0.946	0.847	(0.416, 1.51)	
IIV				
${\omega }_{{\text{slope}}}$(SD)	0.917	0.904	(0.827, 0.978)	–0.46
Unexplained variability				
Additive residual error (*σ*_add_, SD)	0.516	0.512	(0.456, 0.570)	
Proportional residual error (*σ*_pro_, SD)	0.4	0.404	(0.282, 0.487)	

^a^Slope estimate refers to the 3.0% once-daily treatment group in the atopic dermatitis population

*CI*, confidence interval; *IIV*, inter-individual variability; *logLik*, log likelihood; *PsO*, psoriasis; *SD*, standard deviation; *SHK*, shrinkage

**Fig. 3:**
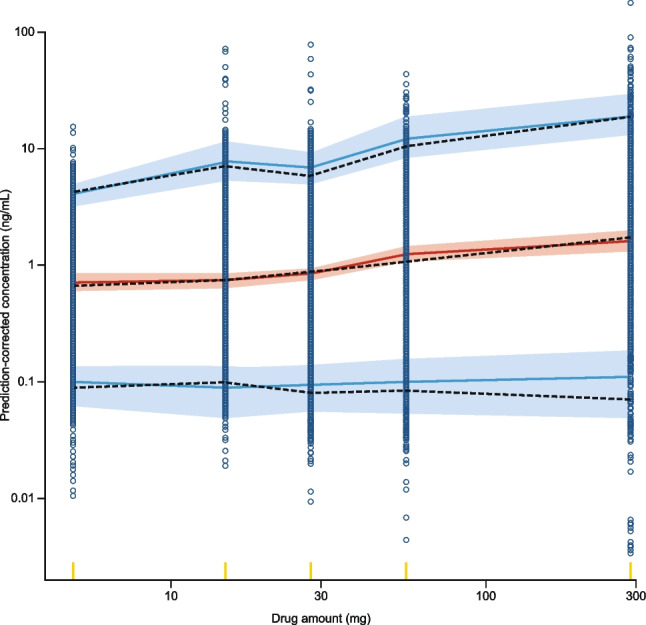
Prediction-corrected visual predictive check. The observed data are represented by the blue circles and dashed black lines (median, 5th, and 95th percentiles). Simulated *C*_Trough_ values based on the index population (*n* = 1,000 simulations) are represented by the red solid line and red shaded ribbon (median and 95% prediction interval of the median, respectively), and the blue solid lines and blue shaded ribbons (median and 95% prediction interval of the 5th and 95th percentiles, respectively). The y- and x-axes are both logarithmic scales. Yellow indicators on the x-axis represent the amount of drug for summarizing the data. Observed and simulated observations below the limit of quantification observations are excluded. *C*_Trough_, trough concentration.

A VPC was performed to assess the predictive performance of the final model based on 1,000 simulations of the original dataset. The pcVPC is presented in Fig. 3. The final model’s predictions overlay the observed data with good agreement.

### Safety Thresholds from Oral Clinical Studies

Using the developed population exposure–response model for PASI scores, clinical trial simulations were performed, and the results indicated that a dose of 10 mg QD for 16 weeks is the lowest practical dose with an effect on PASI75 (proportion of patients achieving at least 75% improvement from baseline PASI) that can be distinguished from placebo. The average concentration (predicted using popPK model) associated with this dose level is 22.2 ng/mL, which was considered to be the minimum systemic pharmacologic activity level.

The exposure–response models of hematopoietic markers estimated that the reticulocyte and neutrophil EC_10_ (10% maximal inhibitory concentration) were 131 and 547 ng/mL, respectively, which were considered tolerable safety thresholds.

The concentration–QT analysis suggested that the placebo-corrected change from baseline QTcF interval slightly passed the threshold of 10 ms (90% CI: 8.57, 10.96) at a geometric mean of *C*_max_ of 549 ng/mL following a 60 mg QD oral dose of brepocitinib.

### Safety Thresholds From Non-Clinical Toxicity Studies

The NOAELs for brepocitinib when administered orally or topically are shown in Table 3.

**Table 3:** Non-Clinical NOAEL

Adverse finding	Species	Study type	NOAEL (ng/mL)
Developmental toxicity (skeletal malformations, decreased fetal viability)	Rabbit	Embryo–fetal development	70
Bone findings (malrotation, impaired use of hindlimbs, small/absent femoral head, microscopic fracture)	Rat	Juvenile toxicity	151
Mortality related to viral infection (suspected porcine cytomegalovirus)	Minipig	9-month topical minipig toxicity	60

*NOAEL*, no observed adverse effect level

### Optimal Treatment Selection

**Fig. 4:**
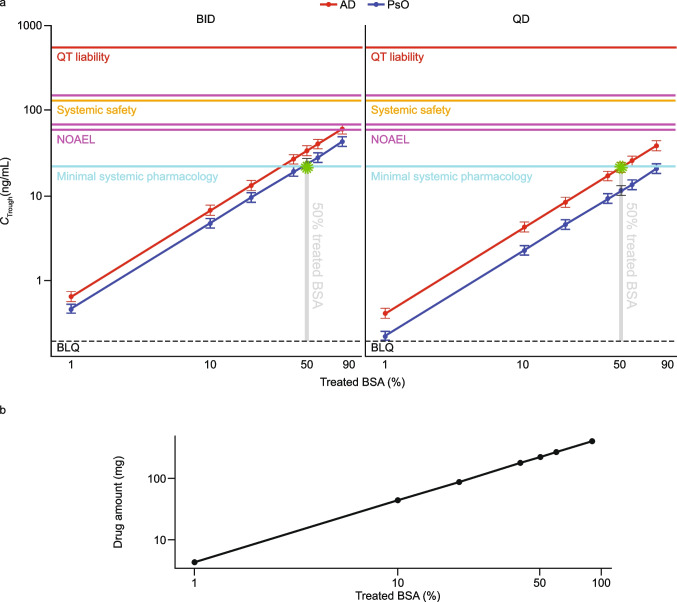
Brepocitinib systemic exposure. (**a**) Points (intervals) representing the projected mean (90% CI) systemic exposure of brepocitinib after topical administration of 3.0% cream in AD and PsO populations with the APR of 2 mg/cm^2^ for different percentage treated BSAs. The y- and x-axes are expressed as a logarithmic scale. (**b**) Black points representing the amount of drug associated with different percentage treated BSAs, calculated using Eq. 2 (see Materials and Methods). The gray dashed line (0.2 ng/mL) is the low limit of quantification; the cyan line (*C*_ave_ = 22.2 ng/mL) is the minimum systemic pharmacologically active level after a 10 mg daily oral dose of brepocitinib. The magenta lines represent the non-clinical NOAEL in the rat juvenile toxicity study (*C*_ave_ = 151 ng/mL), minipig topical toxicity study (*C*_ave_ = 60 ng/mL), and fetal viability in the rabbit EFD study (*C*_ave_ = 70 ng/mL). The orange lines represent the 10% maximal inhibitory concentration in neutrophil (*C*_ave_ = 547 ng/mL) and reticulocyte (*C*_ave_ = 131 ng/mL) counts based on an exposure–response analysis of hematopoietic markers after oral administration of brepocitinib. The red line represents the maximum systemic exposure (*C*_max_ = 547 ng/mL) associated with a 60 mg daily dose of brepocitinib with which the QT liability criteria are violated. The y- and x-axes are expressed as a logarithmic scale. *AD*, atopic dermatitis; *APR*, application rate; *BID*, twice daily; *BLQ*, below the limit of quantification; *BSA*, body surface area; *C*_ave_, average concentration in the dosing interval; *CI*, confidence interval; *C*_Trough_, trough concentration; *EFD*, embryo–fetal development; *NOAEL*, no observed adverse effect level; *PsO*, psoriasis; *QD*, once daily.

**Fig. 5:**
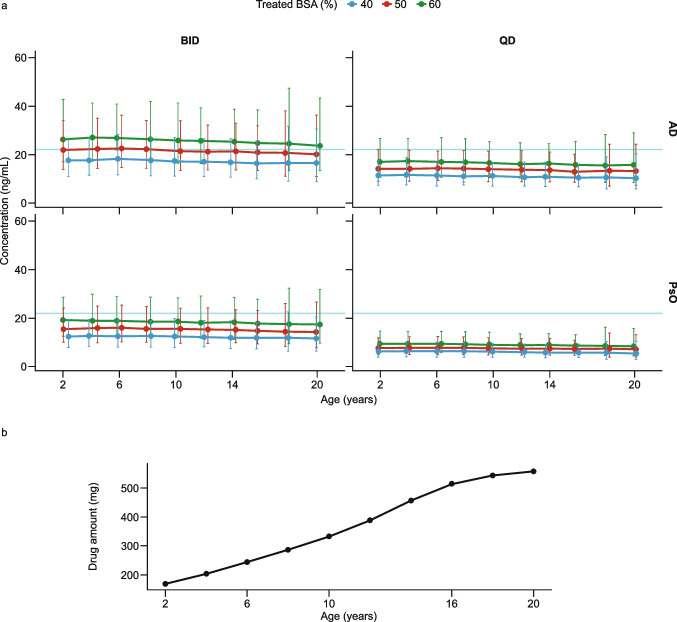
Projection of *C*_Trough_ with 3% topical brepocitinib in a pediatric population and adults up to 20 years of age. (**a**) Projection of systemic *C*_Trough_ for different age groups in AD and PsO patients after topical administration of 3% brepocitinib cream with an APR of 2 mg/cm^2^ for different dosing frequencies. The solid line and error bars represent the median and 90% CI. Colors represent different percentage treated BSAs. As shown, the systemic exposure in pediatric patients down to 2 years of age is comparable with that in adult patients. (**b**) Black points representing the amount of drug associated with different age groups. *AD*, atopic dermatitis; *APR*, application rate; *BID*, twice daily; *BSA*, body surface area; *CI*, confidence interval; *C*_*Trough*_, trough concentration; *PsO*, psoriasis; *QD*, once daily.

- Optimal dose-strength selection: Among all tested dose strengths, the observed topical efficacy data indicated that 3.0% cream demonstrates an optimal balance between efficacy and safety/tolerability; therefore, this dose strength is proposed for evaluation in MUsT and phase 3 studies.
- Optimal regimen selection and maximum treated BSA (%) calculation: The final LMER model was used to perform a clinical trial simulation (number of patients: 220; number of iterations: 1,000) to project systemic exposure (*C*_Trough_) at different percentage treated BSAs (up to 90%) and in different treatment groups in adult patients with AD and PsO, an average BWT of 70 kg, a BSA of 20,000 cm^2^, and an APR of 2 mg/cm^2^ (Table S2 in the Online Resource). As shown in Fig. 4, after topical administration of 3.0% cream in patients with AD following a QD regimen, the maximum treated BSA (%) with which the systemic concentration remained lower than the minimum clinical pharmacologically active level was approximately 50%. The systemic exposure in patients with PsO is expected to be 45% lower than that in the AD population (see Table 2). As shown in Fig. 4, in the PsO population with a BID regimen, the maximum treated BSA (%) with which systemic concentration remained lower than the minimum clinical pharmacology level was also approximately 50%.
- Oral to topical systemic margins calculation: The final model predictions were used to obtain the oral to topical (administration of 3.0% cream with APR of 2 mg/cm^2^) exposure margins using clinical and pre-clinical information. The steady-state systemic exposure after topical application of brepocitinib was expected to be flat over the dosing interval; therefore, *C*_ave_ was assumed to be equal to *C*_Trough_ in the current projection. A summary of oral *C*_ave_ to topical *C*_Trough_ margins is presented in Table 4 for different treated BSAs. The steady-state systemic *C*_Trough_ after topical administration in AD with a QD regimen and PsO with a BID regimen at BSA of 50% are expected to be 22.4 and 21.7 ng/mL, respectively. This level of systemic exposure is considered safe and with minimal systemic pharmacological activity based on the comparison with non-clinical and clinical thresholds.
- Extrapolation of concentrations to pediatric populations: The final model was used to predict systemic *C*_Trough_ in pediatric populations with AD and PsO. Figure 5 shows that systemic exposure of brepocitinib in children was projected to be comparable with that seen in adult patients. This is as expected, based on the first principles of clinical pharmacology, where, for a given percentage treated BSA, the maximum possible dose in a pediatric patient is lower than the dose in an adult patient. Hence, assuming similar topical/transdermal bioavailability, the lower dose offsets the impact of lower clearance in pediatric patients relative to adults (see Eq. 8). This relationship has been demonstrated with crisaborole [27].
- Minimum number of patients per cohort: Sample-size estimation is an important element of clinical trial design, as it is tied to the ability of a trial to detect statistically significant differences [36]. Sample-size estimation helps optimize the number of patients in a trial while maintaining a predefined statistical power. The final LMER model was used to perform a clinical trial simulation based on Table 5, to determine the optimal number of patients to be included in the MUsT with the following assumptions:
  - Threshold = 26.2 ng/mL (95th percentile of *C*_ave_ from 10 mg oral dose based on the PopPK model; this oral dose is associated with minimal systemic pharmacology based on PASI in PsO patients
  - Level of significance = 5%
  - Power = 80%
  - Type of test = two-sided.

**Table 4:** Safety Margins Based on Non-Clinical and Clinical Exposures

Treatment group	Predicted topical *C*_ave_^a^	Margin						
		Non-clinical NOAEL			Safety based on clinical oral program			
		Rat juvenile toxicity^b ^(*C*_ave_ = 151)	Minipig topical toxicity^c^ (*C*_ave_ = 60)	Rat/Rabbit EFD^d^ (*C*_ave_ = 70)	Reticulocyte^e^ (*IC*_10_ = 131)	Neutrophil^e^ (*IC*_10_ = 547)	QT^f^ (*C*_max_ = 549)	Min. systemic pharmacology^g^ (*C*_ave_ = 22)
40% treated BSA								
AD (ng/L)								
3.0% BID	26.5	5.7	2.3	2.6	4.9	20.6	20.7	0.8
3.0% QD	17.0	8.9	3.5	4.1	7.7	32.3	32.4	1.3
PsO (ng/L)								
3.0% BID	18.8	8.0	3.2	3.7	7.0	29.1	29.2	1.2
3.0% QD	9.4	16.0	6.4	7.4	13.9	58	58.2	2.3
50% treated BSA								
AD (ng/L)								
3.0% BID	33.5	4.5	1.8	2.1	3.9	16.3	16.4	0.7
3.0% QD	20.9	7.2	2.9	3.3	6.3	26.1	26.2	1.1
PsO (ng/L)								
3.0% BID	23.8	6.4	2.5	2.9	5.5	23.0	23.1	0.9
3.0% QD	11.8	12.7	5.1	5.9	11.1	46.2	46.3	1.9
60% treated BSA								
AD (ng/L)								
3.0% BID	40.2	3.8	1.5	1.7	3.3	13.6	13.6	0.5
3.0% QD	25.2	6.0	2.4	2.8	5.2	21.7	21.8	0.9
PsO (ng/L)								
3.0% BID	28.3	5.3	2.1	2.5	4.6	19.3	19.4	0.8
3.0% QD	14.1	10.7	4.3	5.0	9.3	38.9	39	1.6

^a^Concentrations are in ng/mL. *C*_ave_ was assumed to be equal to *C*_Trough_ in the current projection. *C*_ave_ in non-clinical studies is calculated from the area under the curve divided by the dosing interval (24 h)

^b^Adverse bone findings in the juvenile rat toxicity study

^c^Mortality related to viral infection in the topical minipig toxicity study

^d^Lower fetal viability in the rabbit EFD study

^e^Based on an exposure–response analysis of hematopoietic markers after administration of brepocitinib

^f^Based on a concentration–QT analysis for brepocitinib

^g^Based on a population pharmacokinetic analysis of brepocitinib

*AD*, atopic dermatitis; *BID*, twice daily; *BSA*, body surface area; *C*_ave_, average concentration in the dosing interval; *C*_*max*_, maximum concentration; *EFD*, embryo–fetal development; *IC*_*10*_, 10% maximal inhibitory concentration; *NOAEL*, no observed adverse effect level; *PsO*, psoriasis; *QD*, once daily

**Table 5:** Simulation Settings for the Sample-Size Estimation

Patient population	Regimen	Age (years)	Application rate (mg/cm^2^)	Treated BSA (%)	No. of patients	No. of simulations
AD	QD	2–18	2	50	8, 10, 12, 14, 16	5,000
PsO	BID					

*AD*, atopic dermatitis; *BID*, twice daily; *BSA*, body surface area; *PsO*, psoriasis; *QD*, once daily

**Fig. 6:**
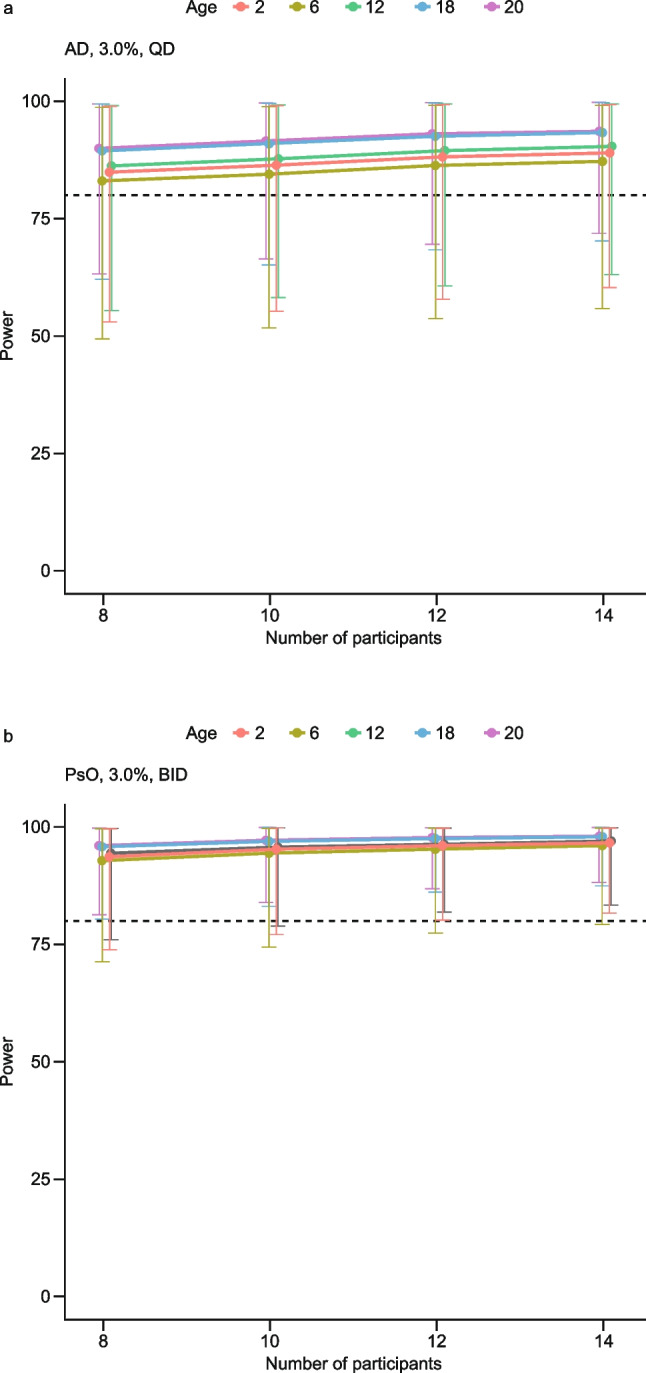
Sample-size estimation. *AD*, atopic dermatitis; *BID*, twice daily; *PsO*, psoriasis; *QD*, once daily.

There is a higher than 80% probability of observing *C*_Trough_ below the 95th percentile of systemic concentration associated with a 10 mg oral daily dose of brepocitinib (i.e., 26.2 ng/mL) with eight evaluable patients per cohort in AD and PsO populations (see Fig. 6).

## Discussion

The objective of this analysis was to characterize the relationship between the amount of topically applied brepocitinib and the resultant systemic exposure, and to use this relationship to inform future clinical trial design in both pediatric and adult populations. This analysis was based on *C*_Trough_ collected in two phase 2 clinical studies in patients with AD and PsO. It should be noted that only trough concentrations were collected, which are not sufficient to develop a longitudinal compartmental population-PK model with absorption and elimination phases, and therefore, these models were not used [27, 37]. An LMER model adequately described the relationship between *C*_Trough_ and the amount of topically applied active drug. Although the amount of active drug was selected as the independent variable, the developed model associates the amount of active drug in cream or the amount of cream with the systemic concentration interchangeably, for ease of communicating future usage instructions. The choice of a linear base model was supported by visual exploration of observed systemic exposures *vs.* drug amount as well as prior knowledge of dose-proportional PK of brepocitinib after a single oral administration up to 100 mg [33]. Systemic exposure of a drug depends on its clearance, which has a non-linear relationship with BWT, and therefore, BWT was included in the model in the form of a power function with a fixed exponent of –0.75 [38]. Inclusion of weight accounted for the difference in clearance across the age range [39]. Covariates such as disease status (AD or PsO), dose strength, and dosing frequency were identified to have significant impacts on the dose–exposure relationship and were therefore included in the final model.

pcVPC demonstrated that the final model adequately predicted the relationship between *C*_Trough_ and the amount of active drug (Fig. 3). Contrary to previous observations with crisaborole where the PK was not different between AD and PsO [27], we predicted the PsO exposure to be 45% lower than that in AD. This difference suggests that the epidermal barrier function of patients with AD treated with topical brepocitinib may have been impacted more than that of patients with PsO and, as a result, patients with AD would have higher apparent clearance due to higher bioavailability. That skin permeability is disrupted in AD is consistent with previous work [40]. Although the reason behind this difference is unknown, it is important to contextualize its magnitude. Patients with AD still maintained a margin of ~ threefold to both non-clinical and hematological safety thresholds, hence, this difference did not lead to a significant erosion in the margins to safety exposure thresholds. The BID exposure was predicted to be 56% higher than that in QD. Therefore, for a given APR, the systemic exposure after topical administration of brepocitinib in AD patients with a QD dosing regimen is expected to be similar to that in PsO patients with a BID dosing regimen (see Table 4). A comparison between the estimated slope for different dose strengths indicated that although the dose strength varies by 30-fold (0.1–3.0%), the estimated slope varies by 7.4-fold. The 3% treatment showed an optimal balance of efficacy and safety/tolerability. For a 70 kg adult patient with a total BSA of 20,000 cm^2^, the steady-state systemic *C*_Trough_ after topical administration of 3% cream in AD with QD regimen, and PsO with a BID regimen at a BSA of 50% (APR of 2 mg/cm^2^), is expected to be 22.4 and 21.7 ng/mL, respectively. This level of systemic exposure is considered safe and with minimal systemic pharmacological activity based on the comparison with non-clinical and clinical thresholds.

## Conclusions

The relationship between the amount of active drug and brepocitinib systemic *C*_Trough_ was adequately described by an LMER model. Patient type, dose strength, and dosing frequency had significant impacts on the relationship between the amount of active drug and *C*_Trough_. On average, the systemic concentration in patients with PsO is predicted to be 45% lower than that in patients with AD. The systemic concentration of brepocitinib with a BID regimen is expected to be on average 56% higher than with QD. With an APR of 2 mg/cm^2^, the systemic steady-state exposure, after topical administration of 3% QD or BID cream applied to less than 50% BSA in patients with AD or PsO, respectively, maintains at least a threefold margin for non-clinical safety findings and a threefold margin for clinical hematological markers. At a similar percentage treated BSA, brepocitinib systemic exposures in the pediatric population are expected to be in a similar range to that of adults after topical administration.

In summary, the proposed modeling approach quantitatively integrated diverse prior knowledge from both non-clinical and clinical studies after either oral or topical application to inform the late-stage development of the topical program by providing the dose strength/BSA recommendation, as well as the sample size for MUsT, phase 3, and pediatric studies, that is expected to provide the optimal benefit–risk profile for patients with AD and PsO. This work not only informed the development strategy for a brepocitinib topical program but also provided a general framework to evaluate the benefit–risk of topical drugs.

### Supplementary Information

Below is the link to the electronic supplementary material.

Supplementary file1 (PDF 781 KB)

## Data Availability

Upon request, and subject to review, Pfizer will provide the data that support the findings of this study. Subject to certain criteria, conditions, and exceptions, Pfizer may also provide access to the related individual de-identified participant data. See https://www.pfizer.com/science/clinical-trials/data-and-results for more information.
