# Supplementary material for: Pharmacokinetic Profile of Brepocitinib with Topical Administration in Atopic Dermatitis and Psoriasis Populations: Strategy to Inform Clinical Trial Design in Adult and Pediatric Populations
Source: Pharm Res. 2024 Mar 22;41(4):623–36. doi: 10.1007/s11095-024-03654-w (PMC11024034; doi:10.1007/s11095-024-03654-w)

Pharmacokinetic Profile of Brepocitinib with Topical Administration in Atopic Dermatitis and Psoriasis Populations: Strategy to Inform Clinical Trial Design in Adult and Pediatric Populations

Farzaneh Maleki[1] · Cheng Chang[1] · Vivek S. Purohit[1] · Timothy Nicholas[1]

[1]Clinical Pharmacology & Pharmacometrics, Global Product Development, Pfizer, Cambridge, MA 02139, USA

Corresponding author

Farzaneh Maleki

Clinical Pharmacology & Pharmacometrics, Global Product Development, Pfizer, Cambridge, MA 02139, USA

farzaneh.maleki@pfizer.com

Keywords atopic dermatitis · brepocitinib · population pharmacokinetics · psoriasis · topical drug

Supplementary Material

Table S1 Summary of Clinical Studies Included in the Analysis

Protocol	Phase	Protocol design	Population	No. of patients	Dose administration	Plasma sampling
B7931022	2	Phase 2b, randomized, double-blind, vehicle-controlled, parallel-group, dose-ranging study to assess the efficacy, safety, tolerability, and PK of brepocitinib cream applied QD or BID for 6 weeks in participants with mild or moderate AD	AD	225	Brepocitinib (topical) 0.1%, 0.3%, 1%, 3%, and vehicle in QD or BID regimen	Days 1, 8, 15, 22, 29, and 43; PK plasma was sampled prior to dosing
B7931023	2	Phase 2b, randomized, double-blind, vehicle-controlled, parallel-group, dose-ranging study to assess efficacy, safety, tolerability, and PK of brepocitinib topical cream applied QD or BID for 12 weeks in participants with mild to moderate chronic PsO	PsO	266	Brepocitinib (topical) 0.1%, 0.3%, 1%, 3%, and vehicle in QD or BID regimen	Days 1, 8, 15, 29, 43, 57, 71, and 85; PK plasma was sampled prior to dosing

AD, atopic dermatitis; BID, twice daily; PK, pharmacokinetics; PsO, psoriasis; QD, once daily

Table S2 Predicted C_{Trough} for Different Percentage Treated BSAs and Treatment Groups in Adults

Treatment group	Predicted topical C_{Trough} (90% CI)				
	Treated BSA (%)				
	10	20	40	50	60
AD					
0.1% BID	1.1 (1.0–1.3)	2.3 (2.0–2.5)	4.5 (4.0–5.1)	5.6 (5.0–6.4)	6.7 (5.9–7.6)
0.1% QD	1.0 (0.9–1.2)	2.1 (1.8–2.4)	4.1 (3.6–4.7)	5.2 (4.6–5.9)	6.3 (5.6–7.1)
0.3% BID	1.9 (1.7–2.2)	3.8 (3.3–4.3)	7.6 (6.8–8.7)	9.6 (8.4–10.9)	11.3 (10.1–12.9)
0.3% QD	1.6 (1.5–1.9)	3.3 (2.9–3.8)	6.6 (5.8–7.6)	8.3 (7.3–9.5)	10.0 (8.8–11.4)
1.0% BID	3.6 (3.1–4.1)	7.1 (6.3–8.1)	14.2 (12.5–16.3)	17.8 (15.7–20.3)	21.4 (18.9–24.3)
1.0% QD	2.7 (2.4–3.1)	5.5 (4.9–6.3)	10.9 (9.7–12.5)	13.9 (12.2–15.7)	16.5 (14.4–18.8)
3.0% BID	6.7 (5.9–7.6)	13.2 (11.7–15.0)	26.5 (23.4–30.0)	33.5 (29.5–38.1)	40.2 (35.5–45.5)
3.0% QD	4.2 (3.7–4.8)	8.5 (7.5–9.6)	17 (14.9–19.1)	20.9 (18.6–24)	25.2 (22.3–28.8)
PsO					
0.1% BID	1.1 (0.9–1.2)	2.1 (1.9–2.4)	4.2 (3.7–4.8)	5.3 (4.7–6.0)	6.4 (5.6–7.3)

0.1% QD	1.0 (0.9–1.1)	2.0 (1.7–2.2)	3.9 (3.4–4.5)	4.9 (4.3–5.6)	5.8 (5.1–6.6)
0.3% BID	1.7 (1.5–1.9)	3.4 (3–3.9)	6.8 (6.0–7.7)	8.6 (7.5–9.7)	10.2 (9.0–11.6)
0.3% QD	1.5 (1.3–1.7)	2.9 (2.5–3.3)	5.9 (5.2–6.7)	7.3 (6.5–8.3)	8.8 (7.8–10.1)
1.0% BID	2.9 (2.6–3.3)	5.8 (5.2–6.6)	11.6 (10.2–13.3)	14.6 (13–16.6)	17.3 (15.3–19.8)
1.0% QD	2.1 (1.9–2.4)	4.2 (3.7–4.8)	8.5 (7.5–9.8)	10.6 (9.3–12.0)	12.7 (11.2–14.4)
3.0% BID	4.7 (4.2–5.4)	9.4 (8.3–10.7)	18.8 (16.8–21.5)	23.8 (20.9–27)	28.3 (24.8–32.2)
3.0% QD	2.3 (2.1–2.7)	4.7 (4.1–5.3)	9.4 (8.3–10.8)	11.8 (10.5–13.4)	14.1 (12.4–16.0)

AD, atopic dermatitis; BID, twice daily; BSA, body surface area; CI, confidence interval; C_{Trough}, systemic trough concentration; PsO, psoriasis; QD, once daily

Monolix Model File

1. Monolix.mlxtran

```
<DATAFILE>

[FILEINFO]
file='B793_Topical_POPPK_Combined.csv'
delimiter = comma
header = {PROJ, PROT, STID, AMT, DOSE, DRUG_mg, BSD, BSD_cm2, BBSA_cm2,
TAFD, DAYP, DV, MDV, EVID, BLQ, SEX, PTST, RACE, TRT, TRTG, Strength,
Regimen, BWT, AGE}

[CONTENT]
STID = {use=identifier}
DRUG_mg = {use=regressor}
TAFD = {use=time}
DV = {use=observation, name=DV, type=continuous}
MDV = {use=missingdependentvariable}
EVID = {use=eventidentifier}
SEX = {use=covariate, type=categorical}
PTST = {use=covariate, type=categorical}
RACE = {use=covariate, type=categorical}
TRTG = {use=covariate, type=categorical}
Strength = {use=covariate, type=categorical}
Regimen = {use=covariate, type=categorical}
BWT = {use=covariate, type=continuous}
AGE = {use=covariate, type=continuous}

<MODEL>

[COVARIATE]
input = {PTST, Regimen, Strength, BWT, RACE, SEX, TRTG, AGE}

PTST = {type=categorical, categories={'Atopic Dematitis', Psoriasis}}
Regimen = {type=categorical, categories={BID, QD}}
```

```
Strength = {type=categorical, categories={'0.1%', '0.3%', '1.0%', '3.0%'}}
RACE = {type=categorical, categories={'African American', Asian, Other, White}}
SEX = {type=categorical, categories={Female, Male}}
TRTG = {type=categorical, categories={'0.1%_QD', '0.3%_BID', '0.3%_QD', '1.0%_BID',
'1.0%_QD', '3.0%_BID', '3.0%_QD'}}

EQUATION:
logtBWT = log(BWT/70)

DEFINITION:
Regimen_Ref =
{
  transform = Regimen,
  categories = {
  G_BID = BID,
  G_QD = QD },
  reference = G_QD
}

Strength_Ref =
{
  transform = Strength,
  categories = {
  'G_0.1%' = {'0.1%'},
  'G_0.3%' = {'0.3%'},
  'G_1.0%' = {'1.0%'},
  'G_3.0%' = {'3.0%'} },
  reference = 'G_3.0%'
}

EQUATION:
logtAGE = log(AGE/42)

[INDIVIDUAL]
input = {slope_pop, omega_slope, PTST, logtBWT, beta_slope_logtBWT, Strength_Ref,
Cov_pop, beta_Cov_PTST_Psoriasis, beta_Cov_Strength_Ref_G_0_1_,
```

beta_Cov_Strength_Ref_G_0_3_, beta_Cov_Strength_Ref_G_1_0_, Regimen_Ref, beta_Cov_Regimen_Ref_G_BID}

PTST = {type=categorical, categories={'Atopic Dematitis', Psoriasis}}

Strength_Ref = {type=categorical, categories={'G_0.1%', 'G_0.3%', 'G_1.0%', 'G_3.0%'}}

Regimen_Ref = {type=categorical, categories={G_BID, G_QD}}

DEFINITION:

slope = {distribution=logNormal, typical=slope_pop, covariate=logtBWT, coefficient=beta_slope_logtBWT, sd=omega_slope}

Cov = {distribution=normal, typical=Cov_pop, covariate={PTST, Strength_Ref, Regimen_Ref}, coefficient={{0, beta_Cov_PTST_Psoriasis}, {beta_Cov_Strength_Ref_G_0_1_, beta_Cov_Strength_Ref_G_0_3_, beta_Cov_Strength_Ref_G_1_0_, 0}, {beta_Cov_Regimen_Ref_G_BID, 0}}, no-variability}

[LONGITUDINAL]

input = {a, b}

file = 'LMER.txt'

DEFINITION:

DV = {distribution=logNormal, prediction=Cc, errorModel=combined2(a, b)}

<FIT>

data = DV

model = DV

`<PARAMETER>`

Cov_pop = {value=1, method=FIXED}

a = {value=0.5179264365624511, method=MLE}

b = {value=0.3995053020114571, method=MLE}

beta_Cov_PTST_Psoriasis = {value=-0.5046529934535124, method=MLE}

beta_Cov_Regimen_Ref_G_BID = {value=0.5011312073719175, method=MLE}

beta_Cov_Strength_Ref_G_0_1_ = {value=5.305179523430101, method=MLE}

beta_Cov_Strength_Ref_G_0_3_ = {value=2.66613251993437, method=MLE}

beta_Cov_Strength_Ref_G_1_0_ = {value=0.797305784667681, method=MLE}

beta_slope_logtBWT = {value=-0.75, method=FIXED}

c = {value=1, method=FIXED}

omega_slope = {value=0.9066170155552606, method=MLE}

slope_pop = {value=0.0256339098201032, method=MLE}

<MONOLIX>

[TASKS]

populationParameters()

individualParameters(method = {conditionalMean, conditionalMode })

fim(method = Linearization)

logLikelihood(method = Linearization)

plotResult(method = {indfits, parameterdistribution, covariancemodeldiagnosis, covariatemodeldiagnosis, obspred, vpc, residualsscatter, residualsdistribution, randomeffects, saemresults })

[SETTINGS]

GLOBAL:

exportpath = 'Monolix'

nbsimulations = 1000

2. Model.txt

[LONGITUDINAL]

input = {Cov,slope,DRUG_mg}

DRUG_mg={use=regressor}

EQUATION:

Cc = Cov*slope *DRUG_mg

OUTPUT:

output = Cc

Fig. S1 Final model diagnostics plots. (**a**) Observed vs. individual predicted systemic exposure. (**b**) Observed vs. population predicted systemic exposure. (**c**) NPDE vs. drug amount. (**d**) NPDE vs. population predicted systemic exposure. The circles are data points, the solid black line is a line with slope 1 or 0, the solid blue line is a Loess smoothed line, and black dashed lines represent NPDE ± 1 standard deviations from the mean. The y- and x-axes are expressed as a logarithmic scale in (**a**) and (**b**). NPDE, normalized prediction distribution errors.

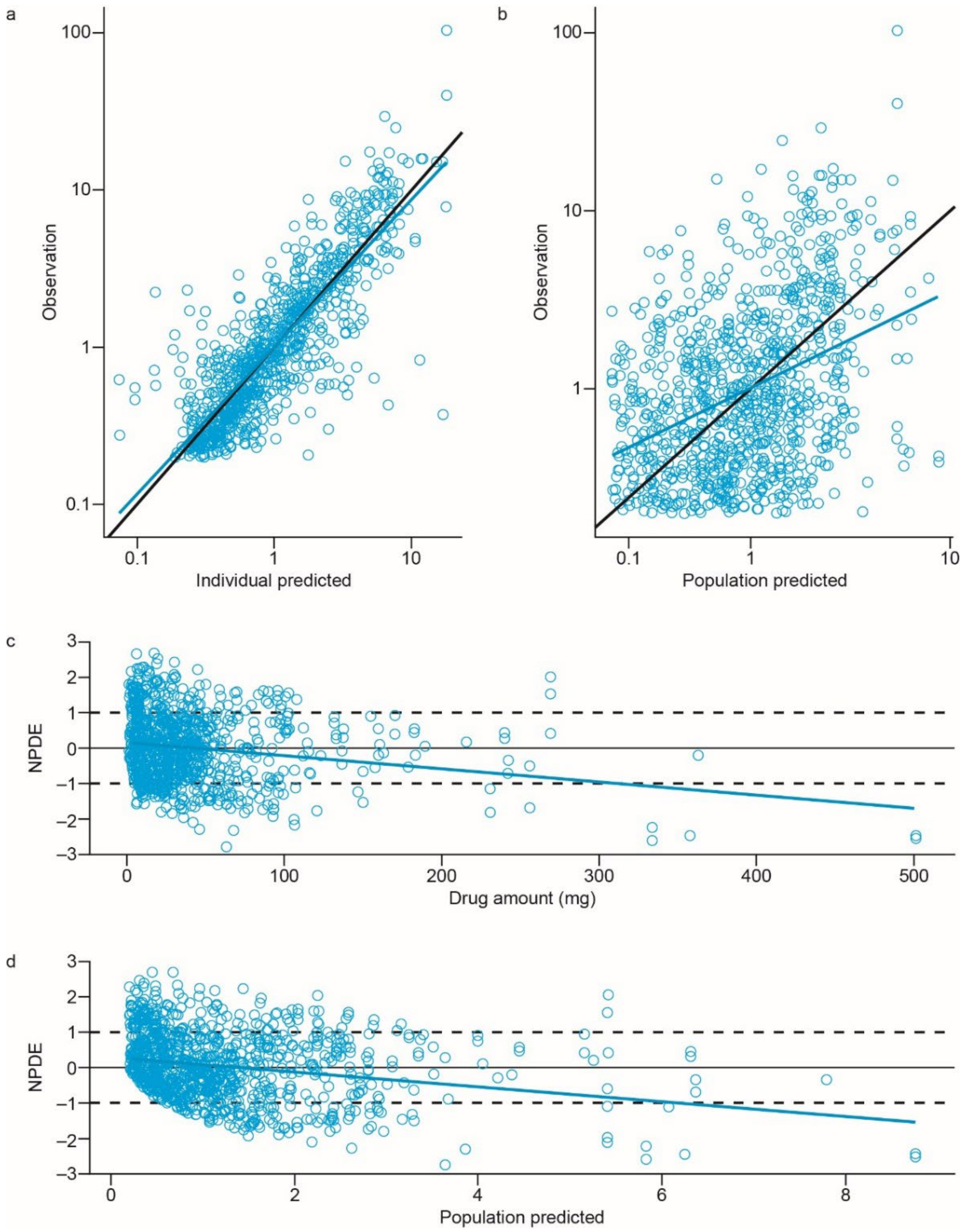

Fig. S2 Final model random-effect distributions. (**a**) Quantile–quantile plot of η. The solid black line is the line of identity. A linear trend is consistent with normality. (**b**) Blue histogram and black solid lines representing the distribution of η, and the black dashed line representing the assumed mean of the η distribution. η_slope, slope is the interindividual variability on the parameter "Slope".

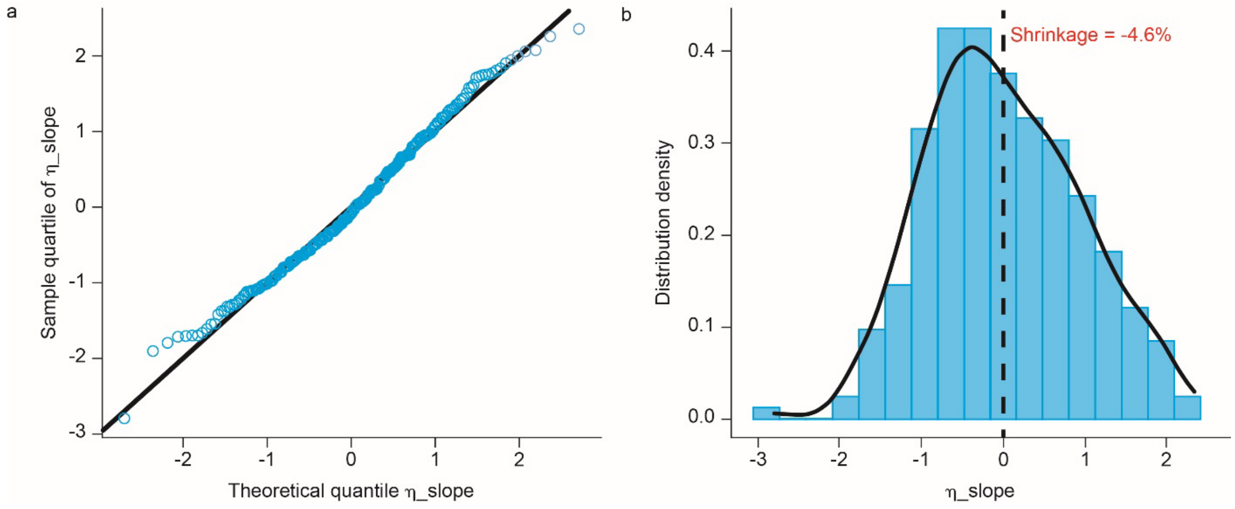

Fig. S3 Final model residual distributions. (**a**) Quantile–quantile plot of NPDE. The solid black line is the line of identity. A linear trend is consistent with normality. (**b**) Blue histogram and solid, black line representing the distribution of NPDE, and the black dashed line representing the assumed mean of the NPDE distribution. NPDE, normalized prediction distribution errors.

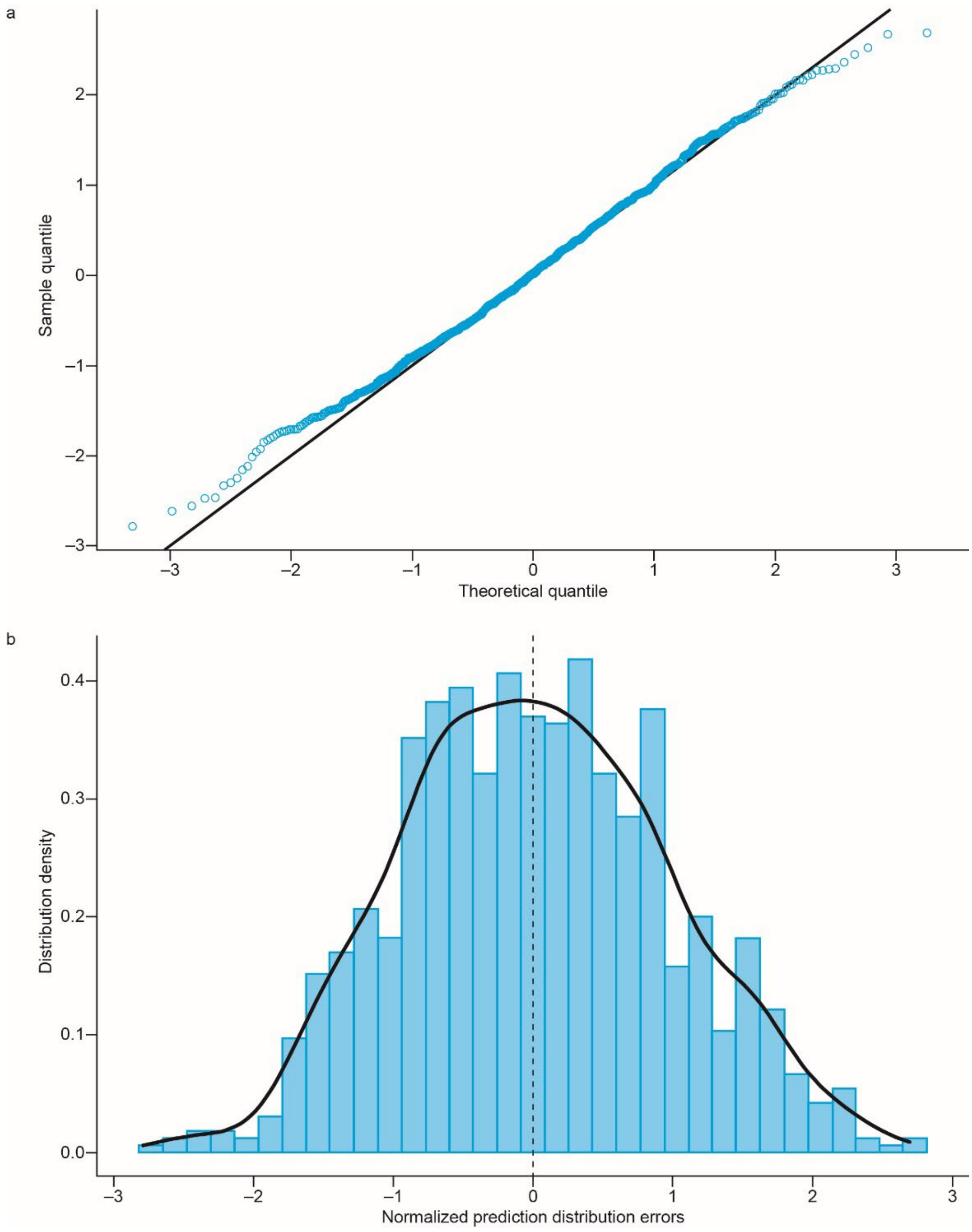

Supplement: Supplementary file 1 — Supplementary file1 (PDF 781 KB) [file 11095_2024_3654_MOESM1_ESM.pdf]
